# Blue Bonds - Antidepressant use during pregnancy and risk of autism spectrum disorder

**DOI:** 10.1192/j.eurpsy.2022.2232

**Published:** 2022-09-01

**Authors:** I. Fonseca Vaz, S. Mouta, B. Jesus, S. Castro

**Affiliations:** Unidade Local de Saúde da Guarda, Departamento De Psiquiatria E Saúde Mental, Guarda, Portugal

**Keywords:** Antidepressants, autism, Pregnancy

## Abstract

**Introduction:**

Epidemiological studies suggested that antidepressant usage amongst pregnant women increased by 3- to 7-fold in the past 2 decades and that up to 13% of fetuses are exposed to antidepressants. There are concerns regarding the impact of prenatal antidepressant use on the offspring.

**Objectives:**

We aim to review more recent evidence of antidepressant use during pregnancy and the risk of autism spectrum disorder (ASD) in the offspring.

**Methods:**

Non-systematic review based on the PubMed® database.

**Results:**

A recent meta-analyses suggested children of mothers who used antidepressants during their pregnancy may be at a higher risk of ASD, compared to mothers who do not use these medications. Other suggested that the use of any antidepressants in the first 2 trimesters was related to significantly risk of developing ASD. Despite higher risks with the use of antidepressants, the absolute risk appears to be low. In addition, confounding factors should be considered while interpreting the results. Studies concluded that the comparator group selection strongly influences the observed antidepressant-ASD relationship. Associations derived from general population studies might have been mediated by unmeasured maternal psychiatric burden or transdiagnostic genetic liability. The observed increase in risk may also be associated with depression, not its treatment.

**Conclusions:**

The findings of these studies have important implications, and often result in drug discontinuation with a significant impact on maternal and infant health. Future research should include investigation of the severity of depression in this association, assessment of antidepressant dose and use of antidepressants in pregnant women with other pathologies.

**Disclosure:**

No significant relationships.

